# CLCA2 overexpression suppresses epithelial-to-mesenchymal transition in cervical cancer cells through inactivation of ERK/JNK/p38-MAPK signaling pathways

**DOI:** 10.1186/s12860-022-00440-7

**Published:** 2022-10-25

**Authors:** Wenhu Xin, Jian Zhang, Haibin Zhang, Xueyao Ma, Yunzhong Zhang, Yufeng Li, Fang Wang

**Affiliations:** 1 Department of Gynecology, Lanzhou University Second Hospital, Lanzhou, 730030 China; 2 The Second Clinical Medical College of Lanzhou University, Lanzhou, 730000 China; 3 Department of Reproductive Medicine, Lanzhou University Second Hospital, No.82, Cuiying Road, Chengguan District, Lanzhou, 730030 China

**Keywords:** CLCA2, p38, Progression, Cervical cancer

## Abstract

Cervical cancer is an important malignant tumor threatening the physical and mental health of women in the world. As a new calcium activated chloride channel protein, calcium activated chloride channel (*CLCA2*) plays an important role in tumorigenesis and development. But its role and exact regulatory mechanism in cervical cancer are still unclear. In our study, we found *CLCA2* was significantly decreased in cervical cancer cells, and overexpression of *CLCA2* inhibited the proliferation, migration and invasion, and promotes apoptosis of cervical cancer cells, and *CLCA2* inhibited EMT (Epithelial-mesenchymal transition) through an *p38 / JNK / ERK* pathway. The results in vivo were consistent with those in vitro. In conclusion, overexpression of *CLCA2* inhibited the progression of cervical cancer in vivo and in vitro. This may provide a theoretical basis for *CLCA2* as a new indicator of clinical diagnosis and prognosis of cervical cancer or as a potential target of drug therapy.

## Introduction

Cervical cancer is the most common malignant tumor in women in China, and it is also a cause of death in women all over the world In recent years, the incidence of cervical cancer has been controlled after early screening and vaccine prevention, but the cure rate has declined [1]. Drug resistance, invasion and metastasis, and postoperative recurrence are the common causes of death in patients with advanced cervical cancer [2]. They are also important factors affecting the treatment and clinical prognosis of patients. Although patients with advanced cervical cancer can not be completely cured, there are many treatments that can improve the length and quality of life of patients. With the development of molecular biology technology and the application of medical field, it is more and more important to carry out molecular biology research on the pathogenesis of cervical cancer, and provide experimental and theoretical basis for clinical prevention and treatment [3–5]. Calcium activated chloride channel (*CLCA*) is one of the chloride channels [6]. It can be widely expressed in various tissues and cells, and participate in a variety of physiological processes, such as sensory conduction, transepithelial ion and fluid secretion, excitation of myocardial cells and nerve cells, and contraction of smooth muscle cells [7]. *CLCA* family is a new type of calcium activated chloride channel family, which mainly includes *CLCA1, CLCA2* and *CLCA4*. The gene is located at the short arm site of chromosome 1 (1p22-31). The upstream fragment of *CLCA2* gene is 5′-AGACAATCCCTACACCCTTCAA-3′, and the downstream fragment is 5′- TGTCGGTAGAACACCTTGTCAC-3′, and the size of its transcript fragment is 233 bp. Among the known family members, *CLCA2* and *CLCA4* have similar structures. They are typical type I transmembrane proteins with a size of about 125 kDa and about 900 amino acids. There are symmetrical cysteine motifs CX12CX4 CX4CX12C at the amino terminal. *CLCA* transmembrane protein can be rapidly cleaved into 90 and 35 kDa subunits and interact with integrin through semi conserved sequences in subunits- β4 interaction to promote early hematogenous metastasis and growth of tumor. In recent years, *CLCA2* has been found to be closely related to a variety of tumors [8]. *CLCA2* has been proved to be closely related to invasion and metastasis of breast cancer. It is often thought to be down regulated in breast cancer, which can induce breast cancer cells to differentiate into normal cells. Knocking out *CLCA2* gene will induce EMT and increase the invasiveness of cancer cells. However, up to now, the regulatory role of CLCA2 in cervical cancer has not been clarified. Our team previously found that there was a significant difference in the expression of CLCA2 in human normal cervical tissues, low-grade squamous intraepithelial lesion (LSIL), high-grade squamous intraepithelial lesion (HSIL) and cervical squamous cell carcinoma tissues by immunohistochemical studies (*P* < 0.05), and speculated that CLCA2 may be involved in the development of cervical cancer.

In this study, we first detected the expression of *CLCA2* in cervical cancer cell lines siha，hela, and C33A. After stable overexpression of *CLCA2* by lentivirus vector, the changes of cell proliferation, apoptosis, migration and invasion were detected, and the changes of EMT related markers and related signaling pathways were detected. Then the tumor formation experiment in nude mice was carried out to verify the results in vitro. This study preliminarily explored the role of *CLCA2* in cervical cancer, in order to provide a certain basis for further clinical application.

## Materials and methods

### Cell culture

C33A(CL-0045), SiHa (CL-0210) and HeLa (CL-0101) cells were obtained from Procell. H8 cells were obtained from Qingqi (Shanghai) Biotechnology Development Co., Ltd. They were removed from liquid nitrogen and quickly put into a 37 °C water bath. After dissolving, the cells were transferred to a centrifuge tube containing 5 ml medium. The cells were collected by centrifugation, centrifuged at 1000 rpm for 5 min at room temperature, and the supernatant was discarded; The cells were suspended in the complete medium containing 10% fetal bovine serum, inoculated into the culture dish, gently blown and mixed, and cultured at 37 °C and 5% CO2 saturation humidity.

### qRT-PCR

Quantitative real-time PCR is a method to measure the total amount of products after each polymerase chain reaction (PCR) cycle with fluorescent chemicals in a DNA amplification reaction. A method for quantitative analysis of specific DNA sequences in samples to be detected by internal or external reference. Total RNA was extracted from cells using TRIzol reagent (Invitrogen, Carlsbad, CA, USA) according to the manufacturer’s instructions. For mRNA detection, first-strand cDNA was synthesized using a RevertAid first strand cDNA synthesis Kit (Thermo Fisher Scientific Inc., Waltham, MA, USA). All cDNA samples were individually configured for real-time quantitative PCR reaction systems. The system was configured as follows: SYBR Green 10 uL, upstream primer 1 ul, downstream primer 1 uL, dNTP 1 uL, Taq polymerase 2 uL, cDNA of the sample to be tested 5 uL, ddH2O 30 uL, total volume 50 uL, the solution was mixed by flicking the bottom of the tube, and briefly centrifuged at 6000 rpm. Place the prepared PCR reaction solution on the Realtime PCR instrument for PCR amplification reaction. The reaction conditions were as follows: predenaturation at 93 °C for 2 minutes, followed by 40 cycles of 93 °C for 1 minute, 55 °C for 1 minute, and 72 °C for 1 minute, with a final extension at 72 °C for 7 minutes. Relative gene expression was calculated automatically using 2-ΔΔCt. The primer sequences were showed in Table 1.

**Table 1:** Primer sequences

Gene	Primer	Sequence (5′-3′)	PCR Products
Homo GAPDH	Forward	TCAAGAAGGTGGTGAAGCAGG	115 bp
	Reverse	TCAAAGGTGGAGGAGTGGGT	
Homo E-cadherin	Forward	CGTAGCAGTGACGAATGTGG	175 bp
	Reverse	CTGGGCAGTGTAGGATGTGA	
Homo N-cadherin	Forward	CTTGCCAGAAAACTCCAGGG	213 bp
	Reverse	TGTGCCCTCAAATGAAACCG	
Homo snail	Forward	ATGCACATCCGAAGCCACA	190 bp
	Reverse	TGACATCTGAGTGGGTCTGG	
Homo twist	Forward	GCGGCCAGGTACATCGACTTCCTCT	141 bp
	Reverse	CATGGACCAGGCCCCCTCCATCCTC	
Homo CLCA2	Forward	AGATGTGCAGCCTCAGAAGT	204 bp
	Reverse	CTGCGGCTTGTTGTAGTTGA	

### Westernblot

Collect protein sample preparation Use cell lysate to lyse adherent cells, suspended cells or tissue samples. Protein concentration was then determined for each protein sample. An appropriate amount of concentrated SDS-PAGE protein loading buffer was added to the collected protein samples. It is generally recommended to use low voltage constant voltage electrophoresis when the upper gel is used, while high voltage constant voltage electrophoresis is used when bromophenol blue enters the lower gel. Electrophoresis is expected to stop once the target protein has been properly resolved. Bio-Rad ‘s standard wet transfer apparatus was used with a transfer current set at 300-400 mA and transfer time of 30-60 minutes. Using Bio-Rad ‘s standard wet transfer apparatus, the transfer current can be set at 300-400 mA and the transfer time 30-60 minutes. Western blocking solution was added and blocked overnight at 4 °C. Phosphorylated protein was closed with 1% BSA. And then incubated overnight at 4 °C with mouse anti-human *E-cadherin*(1:5000, Sanying, Wuhan, CN), *N-cadherin*(1:5000, Sanying, Wuhan, CN), *twist*(1:1000, Abcam, Cambridge, MA, USA) and rabbit anti-human *GAPDH*(1:1000, Cell Signaling Technology, Danvers, MA, USA), *snail*(1:1000, Bioss, Woburn, MA, USA), *p-p38*(1:1000, Affinity Biosciences, OH, USA), *p-JNK*(1:1000, Cell Signaling Technology, Danvers, MA, USA), *p-ERK*(1:1000, Affinity Biosciences, OH, USA), *p38*(1:1000, Cell Signaling Technology, Danvers, MA, USA), *JNK*(1:1000, Cell Signaling Technology, Danvers, MA, USA), *ERK*(1:1000, Cell Signaling Technology, Danvers, MA, USA). After washing with TBST, the blots were incubated with horse radish peroxidase (HRP)-conjugated goat anti-rabbit or anti-mouse IgG(1:2000, Boshide, Wuhan, CN). Blots were visualized using ECL reagents (Pierce, Rockford, IL, USA) by a chemiluminescence imaging system (Bio-Rad, Richmond, CA, USA).

### Lentivirus infection experiment

The human vector pLVX-mCMV-ZsGreen-IRES-Puro was obtained from Vinoser Biotechnology Co., Ltd. (Wuhan, CN). Human *CLCA2* gene was amplified by PCR using plasmid CLCA2 as template. EcoRI and BamHI sites were introduced at both ends of primers. The connection between the large fragment of plasmid pLVX-mCMV-ZsGreen-IRES-Puro and human *CLCA2* fragment, the ligation reaction was carried out at 22 °C for 3 hours. Take 10ul ligation product and 100ul JM109 competent bacteria, mix them in ice bath for 30 min, heat shock at 42 °C for 60s, put them on ice immediately for 2 min, add 400ul LB medium preheated to room temperature, culture them in constant temperature shaker at 37 °C for 1 h, centrifuge at 4000 rpm for 3 min, discard 400ul culture supernatant, mix the remaining 100ul with pipette, and evenly coat them on LB plate containing 100μg / ml ampicillin resistance, The cells were cultured upside down in 37 °C incubator overnight. Three single colonies were selected and inoculated in the medium containing 5 ml, 100 μg/ml ampicillin resistant LB medium. The plasmids were digested with EcoRI + BamHI, and the correct clones were selected for sequencing. The cell culture medium was changed and virus was added to infect the cells. After mixing, the cells were cultured continuously. After 8-12 hours, the cells were observed and replaced with fresh medium. After 3-4 days of infection, the fluorescence expression was observed, and the medium could be changed midway to maintain the cell activity. The infection conditions and parameters of target cells were confirmed by observing the effect of cell infection.

### Colony formation assay

SiHa, HeLa and C33A were made into single cell suspension by DMEM, 2 × 10^5^ cells were evenly seeded into the 6-well plate; Under the condition of 37 °C and 5% CO_2_ saturation humidity, the cells were cultured for 48 h; According to the titer of lentivirus, lentivirus was added to treat the cells for 72 hours. After lentivirus treatment, the cells were digested with 0.25% trypsin and blown into single cells. The cells were suspended in the medium for standby; The cell suspension was diluted and seeded in a 6-well plate with a density of 300 cells; Turn it gently to make the cells disperse evenly; The culture dish was placed in a 5% CO2 incubator at 37 °C for 2-3 weeks; When the clone appeared, the supernatant was discarded, washed with PBS twice and fixed with 70% ethanol for 15 min; Discard the fixed solution and add appropriate amount of crystal violet staining solution for 10-30 min; Clean with PBS and count.

### Transwell assay

Normal cells, NC (negative control) and -ex*CLCA2* lentivirus treated cells were added with 3 mL PBS to clean the cells, digested and collected with 0.25% trypsin, centrifuged at 1000 rpm for 5 min, supernatant was removed, and PBS was used to wash the residual serum twice; The cells were resuspended in serum-free DMEM medium and counted by cell counting plate. The cell concentration was diluted to 2.5 × 105 cells/mL in serum-free DMEM medium. 200uL cell suspension was added into the Transwell chamber, and cultured in 5% CO2 incubator at 37 °C; The Transwell was removed, the chamber was carefully cleaned with PBS, and the cells were fixed with 70% ice ethanol solution for 1 h; The cells were stained with 0.5% crystal violet dye, placed in room temperature for 20 minutes, PBS was cleaned, the non moving cells on the side of the upper chamber were wiped with clean cotton balls, and the microscope was observed and photographed.

### Flow cytometry

The treated cells were washed with PBS twice, centrifuged at 1000 rpm for 5 min, and detected by flow cytometry according to the operation instructions of AnnexinV-APC/7-AAD cell apoptosis detection kit (Keji biology, Nanjing, CN). Joined 500 μL binding buffer, resuspend cells; Added 5 μL AnnexinV-APC, mix well and add 5 μL 7-AAD, mix well; Room temperature photoprotection reaction for 5-15 min (negative control was set at the same time, that is, normal cells were not added with annexin AnnexinV-APC and 7-AAD). Finally, flow cytometry was used for detection.

### Immunofluorescence

In the culture plate, the glass slide was soaked with PBS for 3 times, 3 minutes each time; The slide was fixed with 4% paraformaldehyde for 15 min, and the slide was soaked with PBS for 3 min each time; 0.5% Triton X-100 (prepared with PBS) was permeable for 20 minutes at room temperature (E-cad and n-cad were permeable for 5 minutes); The slides were soaked in PBS for 3 times, 3 minutes each time. The PBS was sucked dry with absorbent paper. Normal goat serum was dripped onto the slides and sealed at room temperature for 30 minutes; Each slide was dripped with enough diluted primary antibody and put into a wet box, and incubated at 4 °C overnight. Add fluorescent secondary antibody: PBST was used to soak the slides for 3 times, 3 minutes each time. After absorbing the excess liquid on the slides with absorbent paper, the diluted fluorescent secondary antibody was dripped and incubated in a wet box at 37 °C for 1 hour. PBST was used to soak the slices for 3 times, 3 minutes each time; DAPI was dripped and incubated in dark for 5 minutes. The specimens were stained with nucleus and PBST for 5 minutes. The redundant DAPI was washed off 4 times; Water absorbent paper was used to suck up the liquid on the climbing sheet, and the sealing liquid containing anti fluorescence quenching agent was used to seal the sheet, and then the images were observed and collected under the fluorescence microscope.

### Animal study

The SPF female nude mice were purchased from Changzhou cavens experimental animal Co., Ltd., weighing 18-21 g. The animals were divided into normal group, control group and overexpression group, each group had 3 nude mice, and the injected C33A cell volume was 5 × 10 [6]. Gently twist the skin under the right armpit with the left hand, hold a 1 ml syringe parallel to the skin with the right hand, insert the needle into the subcutaneous 1 mL, shake the needle from left to right, there is no resistance and see the needle move in the subcutaneous, indicating that the needle is in the subcutaneous. At this time, inject 100ul tumor cells into the subcutaneous, and gently press the needle eye position for a few seconds after withdrawing the needle to prevent the cells from flowing out. 28 days later, after anesthesia with intraperitoneal injection of 62.5 mg/kg pentobarbital sodium, the animals were sacrificed by dislocation and uniformly sent to Gansu Critically Ill Center for centralized treatment, and half of the tumor tissues were fixed and half frozen for follow-up study.

### Apoptosis was detected by TUNEL

The paraffin embedded tissue sections were put into the staining VAT, and washed twice with xylene for 5 minutes each time. Three minutes each time with anhydrous ethanol, washed twice. Wash with 95 and 75% ethanol for 3 minutes each time. Wash with PBS for 5 minutes. Add proteinase K solution，at 37 °C reaction for 15 min to remove tissue protein. PBS 5 min each time, wash 3 times. PBS containing 3% H_2_O_2_ was added to the dyeing vat and placed at room temperature for 10 minutes. Wash with PBS 3 times, 5 minutes each time. After the excess liquid around the tissue section was carefully removed with filter paper, 2 drops of TdT enzyme buffer were quickly added to the section and placed at room temperature for 1-5 minutes. After carefully absorbing the excess liquid around the slice with filter paper, add 54 uL TdT enzyme reaction solution was put on the slices. Place the sections in the staining jar, add the washing and stopping reaction buffer that has been preheated to 37 °C, keep the temperature at 37 °C for 30 min, gently lift and put down the glass slide every 10 min, so that the liquid is slightly stirred. The sections were washed with PBS three times for 5 minutes each time. Two drops of streptavidin HRP working solution were added directly to the sections and placed in a wet box, reaction in dark for 30 min. Wash with PBS 3 times, 5 minutes each time. The newly prepared 0.05% DAB solution was added to the tissue sections and incubated at room temperature for 30s-5min. Wash with distilled water for 4 times, the first three times for 1 min each time, and the last time for 5 min. After 10 minutes of re staining with hematoxylin at room temperature, the slide was lifted up and put down in distilled water for 20 times, and then left standing for 30s. They were soaked with 70, 85, 95% absolute alcohol for 5 minutes. Xylene was dehydrated 2 min each time for 3 times. Then the film was sealed, dried and photographed under the microscope.

### Statistical analysis

All experiments were performed at least in triplicate, and each experiment was independently performed at least 3 times. The graphical presentations were performed using GraphPad Prism 5.0. Data were presented as the means±SE and were analyzed using SPSS 21.0 software (Chicago, IL, USA). Statistical differences were tested by Chi-square test or two-tailed t-test. Differences were considered significant at *P* < 0.05 (*) or highly significant at *P* < 0.001 (**).

## Results

### Decreased expression of *CLCA2* in cervical cancer cells

**Fig. 1:**
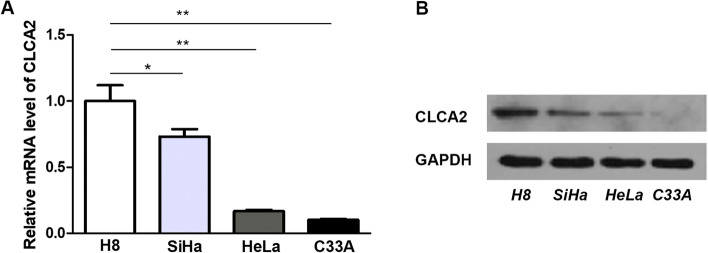
Decreased expression of CLCA2 in cervical cancer cells. **A** qRT-PCR results indicated that the expression of CLCA2 decreased in cervical cancer cells. **B** Westernblot results showed that the expression of CLCA2 decreased in cervical cancer cells. *P < 0.05, **P < 0.001, *t*-test

We first detected the mRNA and protein levels of *CLCA2* in normal cervical cell line H8 and cervical cancer cell lines SiHa, HeLa and C33A. The results showed that the mRNA (Fig. 1A) and protein expression (Fig. 1B) of *CLCA2* in cervical cancer cells was significantly decreased, especially in C33A cell line.

### Overexpression of *CLCA2* inhibits the proliferation, migration and invasion, and promotes apoptosis of cervical cancer cells

**Fig. 2:**
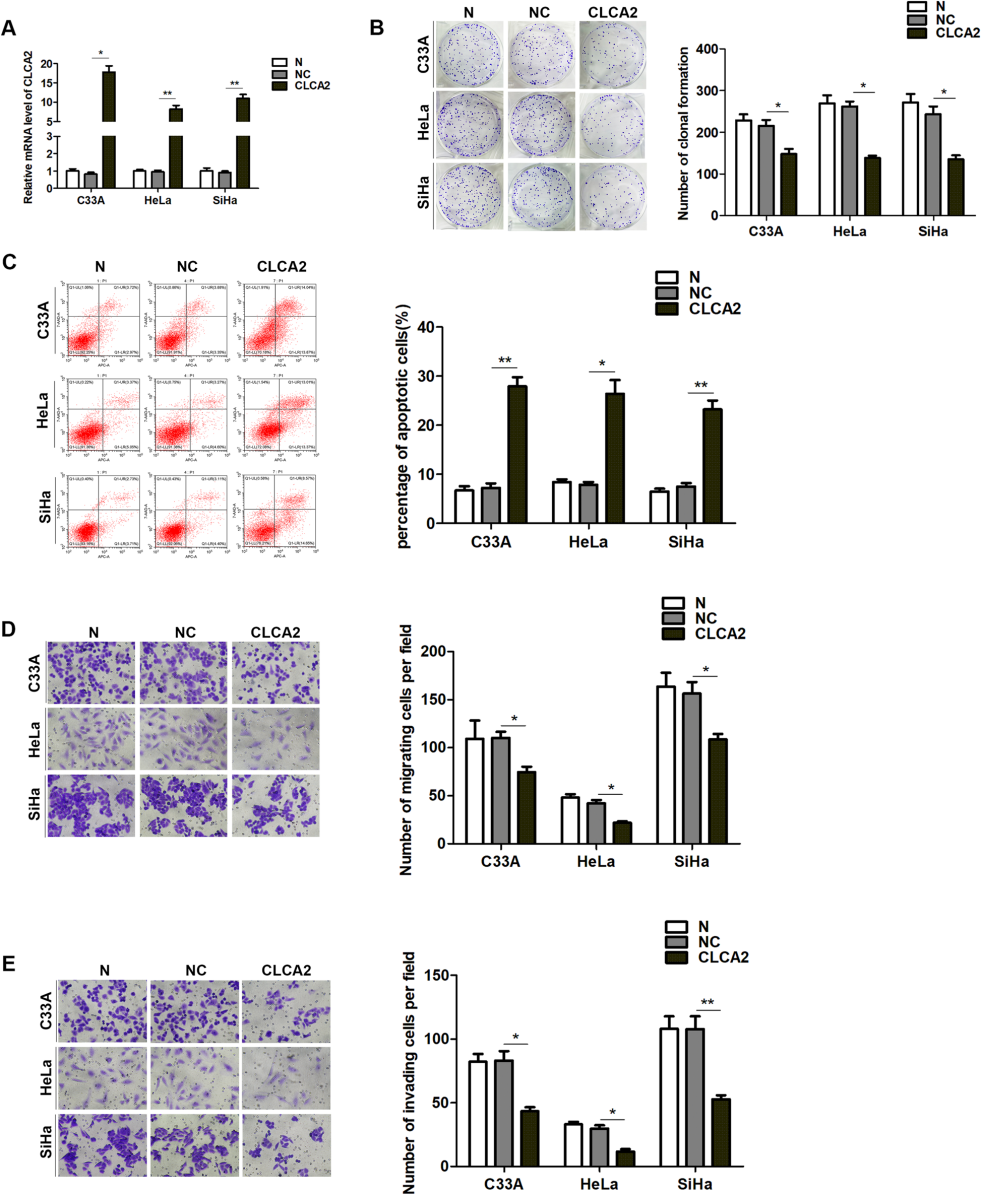
Overexpression of CLCA2 inhibits the proliferation, migration and invasion, and promotes apoptosis of cervical cancer cells. **A** qRT-PCR results indicated that the expression of CLCA2 increased after CLCA2 lentiviral plasmid transfection. **B** Overexpression of CLCA2 inhibited the proliferation of cervical cancer cells. **C** Overexpression of CLCA2 promoted apoptosis of cervical cancer cells. **D** Overexpression of CLCA2 inhibited the migration of cervical cancer cells. **E** Overexpression of CLCA2 inhibited the invasion of cervical cancer cells. *P < 0.05, **P < 0.001, *t*-test. N blank control, NC negative control. Scale bar = 25um

After overexpression of *CLCA2* in cervical cancer cells (Fig. 2A), cell proliferation, migration, invasion and apoptosis were detected. The results showed that overexpression of *CLCA2* inhibited the clonal formation (Fig. 2B), migration (Fig. 2D) and invasion (Fig. 2E), and promoted apoptosis (Fig. 2C) in SiHa, HeLa and C33A cell lines.

### *CLCA2* inhibits EMT in cervical cancer cells

**Fig. 3:**
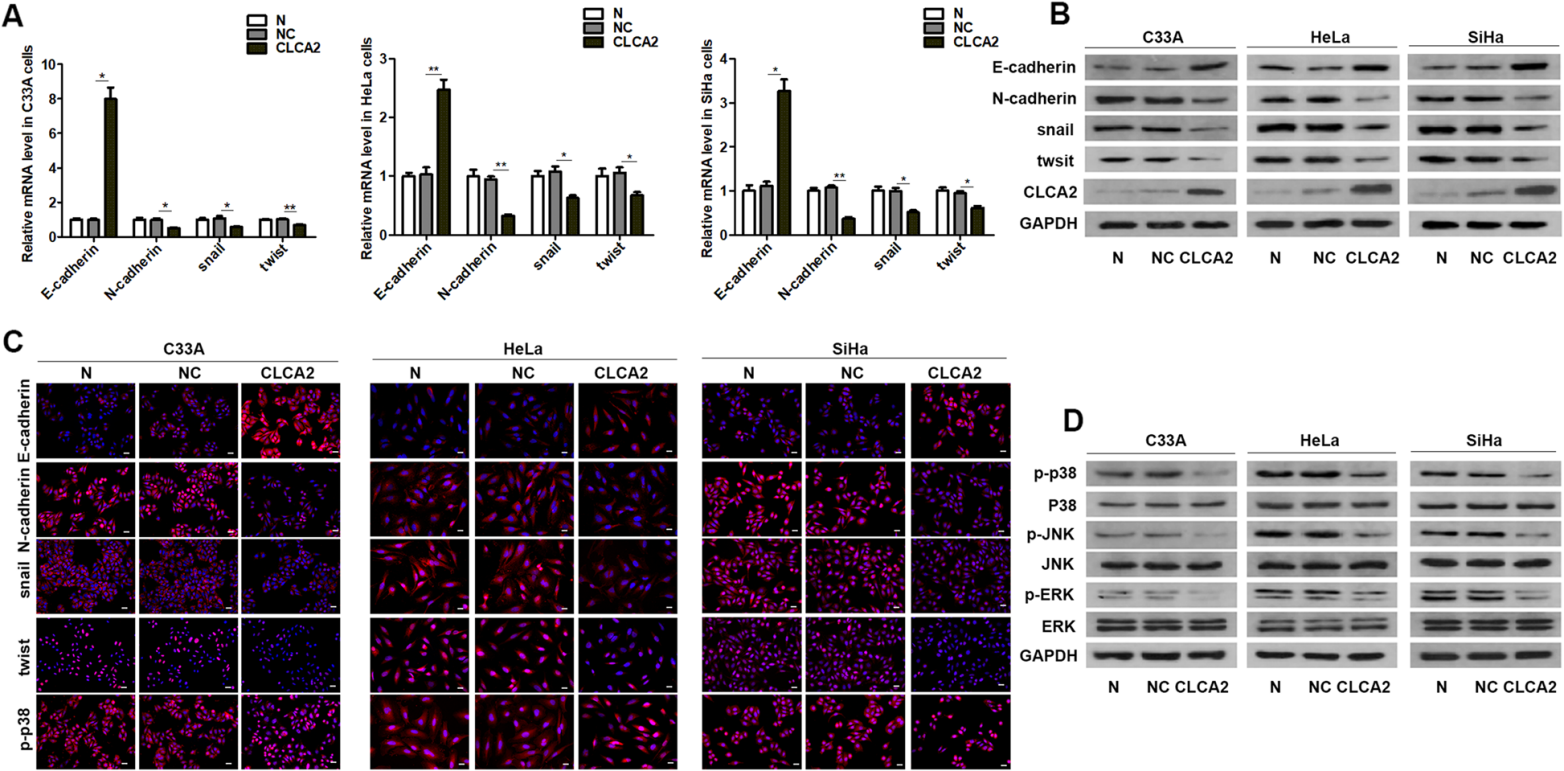
CLCA2 inhibits EMT and p38 / JNK / ERK pathway in cervical cancer cells. Overexpression of CLCA2 up-regulated the expression of E-cadherin and down-regulated the expression of N-cadherin, snail, and twist. **A** qRT-PCR; **B** Westernblot; **C** Immunofluorescence(400×). **D** Westernblot results showed CLCA2 inhibited the expression of p-p38, p-JNK, p-ERK, and the expression level of p38, JNK, and ERK remained unchanged. *P < 0.05, **P < 0.001, *t*-test. Scale bar = 25um

Then, we detected the changes of EMT related markers after overexpression of *CLCA2* in cervical cancer cells. We found that overexpression of *CLCA2* up-regulated the mRNA and protein expression of *E-cadherin* and down-regulated the expression of *N-cadherin* (Fig. 3A, B). *Snail* and *twist* are important transcription factors in EMT. The results showed that overexpression of *CLCA2* significantly decreased the expression of *snail* and *twist* (Fig. 3A, B). Immunofluorescence results showed that overexpression of *CLCA2* could increase *E-cadherin* and decrease *N-cadherin, snail*, *twist*, and *p-p38*(Fig. 3C). These results demonstrated that *CLCA2* inhibits EMT in cervical cancer cells.

### Overexpression of *CLCA2* inhibits *p38 / JNK / ERK* pathway

Previous studies have shown that *CLCA2* can regulate *p38 / JNK / ERK* signaling pathway [9]. We examined the effect of *CLCA2* on the *p38 / JNK / ERK* pathway, the results showed *CLCA2* inhibited the expression of *p-p38, p-JNK, p-ERK*, and the expression level of *p38, JNK,* and *ERK* remained unchanged (Fig. 3D). These results showed overexpression of *CLCA2* inhibits *p38 / JNK / ERK* pathway.

### *CLCA2* inhibits ocervical cancer growth and EMT in vivo

**Fig. 4:**
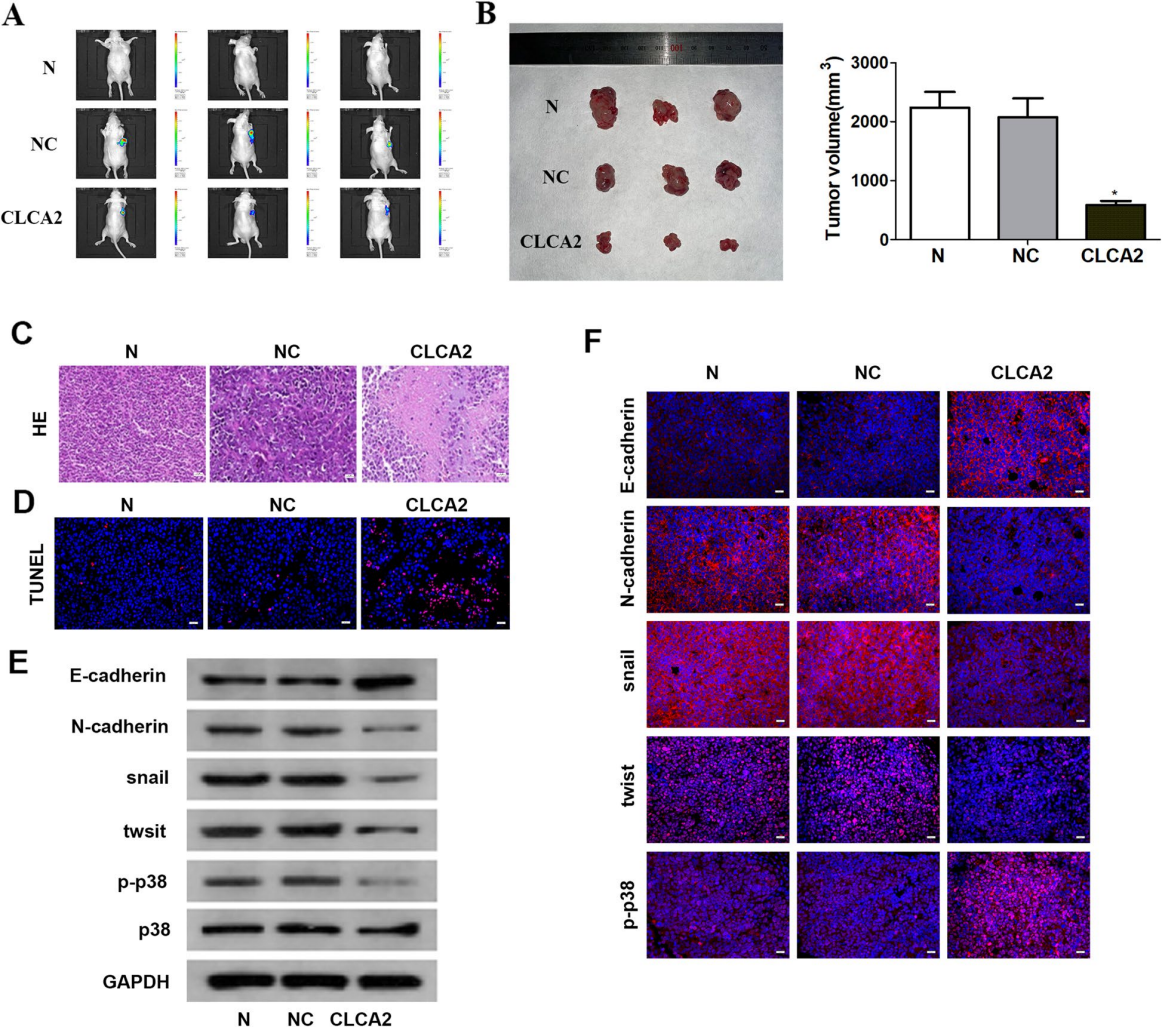
CLCA2 inhibits ocervical cancer growth and EMT in vivo. **A**, **B** CLCA2 overexpression led to a obvious reduction in tumor volume. **C** HE staining showed that the tumor cells had nuclear pyknosis and apoptosis. **D** TUNEL staining showed TUNEL positive cells were observed in tumors from CLCA2 overexpression treatment than those from NC treatment. **E** Immunofluorescence analysis of E-cadherin, N-cadherin, snail, twist and p-p38. **F** Westernblot analysis of E-cadherin, N-cadherin, snail, twist and p38. Scale bar = 100 μm, *P < 0.05, **P < 0.001, *t*-test. Scale bar = 25um

To further validate the effect of *CLCA2* on the growth of cervical cancer cells in vivo, we established a C33A cell tumorigenic model. Consistent with previous observations, *CLCA2* overexpression led to a obvious reduction in tumor volume (Fig. 4A, B). Regarding apoptosis, more TUNEL positive cells were observed in tumors from *CLCA2* overexpression treatment than those from NC treatment (Fig. 4C, D). To further investigate whether *CLCA2* could transform the mesenchymal traits of C33A cells into epithelial phenotypes in vivo, the subcutaneous tumors were fixated for confirmation by immunofluorescence analysis of *E-cadherin, N-cadherin, snail, twist* and *p-p38*, subcutaneous tumors in *CLCA2* expression mice exhibited a significantly high level of *E-cadherin*, and a low level of *N-cadherin, snail, twist.* And *CLCA2* could also inhibit the nuclear translocation of *p-p38 *(Fig. 4F). The result of westernblot was consistent with that of immunofluorescence (Fig. 4E). These in vivo findings coincided with the in vitro changes observed in the cell models, demonstrating that *CLCA2* inhibits cervical cancer growth and EMT.

## Discussion

Cervical cancer is an important malignant tumor threatening the physical and mental health of women in the world, and has a high mortality rate [10]. Therefore, exploring the mechanism of invasion and metastasis of cervical cancer cells is very important for the treatment of cervical cancer and improving the prognosis of cervical cancer patients.

**Fig. 5:**
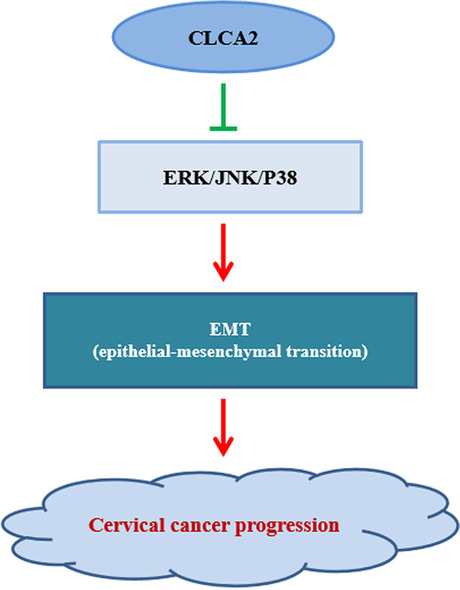
Schematic representation of the mechanism with antitumor effect of CLCA2

At present, little is known about the role of ion channels in tumorigenesis and development, especially the role of calcium activated chloride channel proteins in growth regulation, apoptosis, cell invasion or metastasis. *CLCA2* is a member of calcium activated chloride channel family [11]. It is reported that *NDRG1* gene may play an important role in the invasion and metastasis of cervical and ovarian cancer through its downstream *CLCA2* gene [12]. However, the regulatory role of *CLCA2* in cervical cancer has not been studied. In our study, we found the expression of *CLCA2* in cervical cancer cells was significantly decreased, and overexpression of *CLCA2* inhibits the proliferation, migration and invasion, and promotes apoptosis of cervical cancer cells (Fig. 5).

At present, it is generally believed that epithelial mesenchymal transition (EMT) occurs in epithelial cell-derived malignant tumors. It is generally believed that the central link of EMT is the decrease of E-cadherin expression in epithelial phenotype and the increase of N-cadherin expression in interstitial phenotype [13–15]. *CLCA2* blocked EMT in many cancers [16–19]. In this study, we found that overexpression of *CLCA2* up-regulated the expression of *E-cadherin* and down-regulated the expression of *N-cadherin,* indicate that overexpression of *CLCA2* gene can block epithelial mesenchymal transition (EMT) in cervical cancer cells.

The activation of *ERK* signaling pathway can promote cell proliferation, while the activation of *JNK* signaling pathway is closely related to cell apoptosis, and they are also involved in the pathophysiological changes of many diseases [20–22]. It has confirmed that *CLCA2* can regulate cell function through *p38 / JNK / ERK* pathway [9], and our results also show that *CLCA2* can also regulate the pathway in cervical cancer cells, but the specific mechanism still needs to be further clarified.

As a new calcium activated chloride channel protein, *CLCA2* plays an important role in tumorigenesis and development. Most studies have shown that there are abnormal expression levels and related signaling pathways of *CLCA2* in malignant tumors, and it is closely related to tumor invasion and metastasis [7, 23, 24], *CLCA2* is down regulated in cervical cancer [25, 26], but its role and exact regulatory mechanism in cervical cancer are still unclear. Our results confirmed that the expression of *CLCA2* was significantly decreased in cervical cancer cells. Overexpression of *CLCA2* inhibited the progression of cervical cancer in vivo and in vitro. This may provide a theoretical basis for *CLCA2* as a new indicator of clinical diagnosis and prognosis of cervical cancer or as a potential target of drug therapy.

## Data Availability

All data generated or analysed during this study are included in this published article.

## Abbreviations

CLCA2: Calcium activated chloride channel-2
EMT: Epithelial-mesenchymal transition
JNK: c-Jun N-terminal kinase
ERK: Extracellular regulated protein kinases
